# Supplementary material for: Association between oral microbiome and five types of respiratory infections: a two-sample Mendelian randomization study in east Asian population
Source: Front Microbiol. 2024 Apr 10;15:1392473. doi: 10.3389/fmicb.2024.1392473 (PMC11039966; doi:10.3389/fmicb.2024.1392473)
Supplement: Supplementary file 1 [file Data_Sheet_1.zip › Supplementary Materials/MR plots of saliva/Chronic sinusitis/p__Patescibacteria/scatter.pdf]

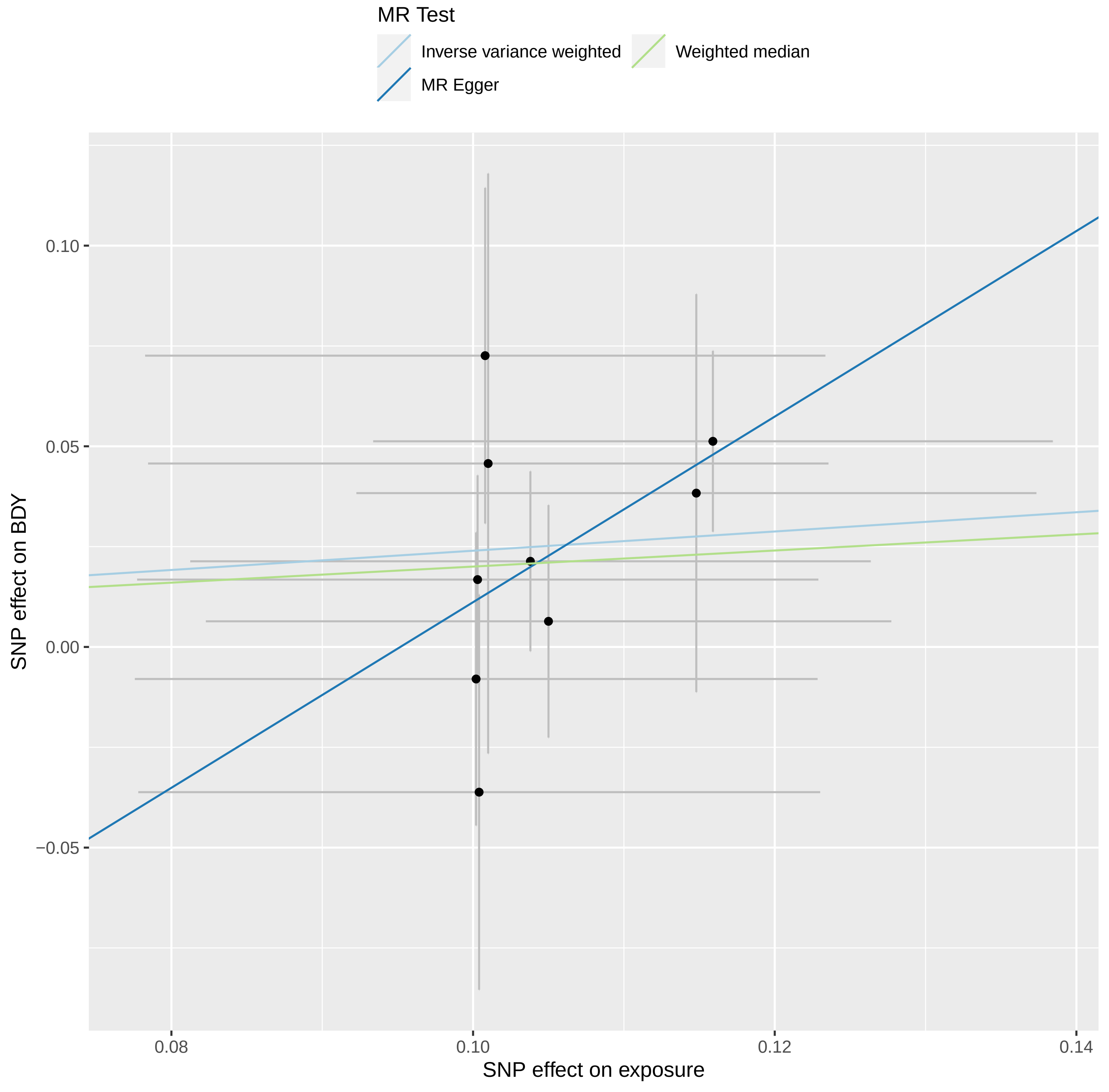
MR Test
Inverse variance weighted
Weighted median
MR Egger
SNP effect on BDY
0.10
0.05
0.00
−0.05
0.08
0.10
0.12
0.14
SNP effect on exposure
